# The Primary Physiological Roles of Autoinducer 2 in *Escherichia coli* Are Chemotaxis and Biofilm Formation

**DOI:** 10.3390/microorganisms9020386

**Published:** 2021-02-14

**Authors:** Sooyeon Song, Thomas K. Wood

**Affiliations:** 1 Department of Animal Science, Jeonbuk National University, Jeonju-si 54896, Jeollabuk-do, Korea; songsy@jbnu.ac.kr; 2 Department of Chemical Engineering, Pennsylvania State University, University Park, PA 16802-7000, USA

**Keywords:** AI-2, chemotaxis, aggregation, biofilm, motility, *Escherichia coli*

## Abstract

Autoinducer 2 (AI-2) is a ubiquitous metabolite but, instead of acting as a “universal signal,” relatively few phenotypes have been associated with it, and many scientists believe AI-2 is often a metabolic byproduct rather than a signal. Here, the aim is to present evidence that AI-2 influences both biofilm formation and motility (swarming and chemotaxis), using *Escherichia coli* as the model system, to establish AI-2 as a true signal with an important physiological role in this bacterium. In addition, AI-2 signaling is compared to the other primary signal of *E. coli*, indole, and it is shown that they have opposite effects on biofilm formation and virulence.

## 1. Introduction

Quorum sensing (QS) is the process by which bacteria communicate via secreted signals (autoinducers); once the concentration of the autoinducers reaches a threshold, the signal is detected, and gene expression is altered [1]. The roles of QS are diverse and include population density detection, virulence, biofilm formation, and the maintenance of the stress response [2]. Although inhibitors of QS (quorum-quenching compounds) are still promoted as a means to reduce virulence without promoting resistance [3], these compounds will indubitably and unfortunately fail. The main problem is that the inhibition of QS leads to pleiotropic effects that affect growth; hence, lab strains and clinical isolates rapidly evolve resistance to these compounds [4,5,6]. Clearly, it is imperative to have a better understanding of QS in order to be in a position to better control bacteria to prevent diseases, such as stomach cancer and ulcers caused by *Helicobacter pylori* and Lyme disease by *Borrelia burgdorferi* [7], and to utilize them for synthetic biology applications. Therefore, in this opinion piece, we probe the physiological role of AI-2 by focusing on the best-studied bacterium, *Escherichia coli.*

## 2. Autoinducer-2

**Figure 1:**
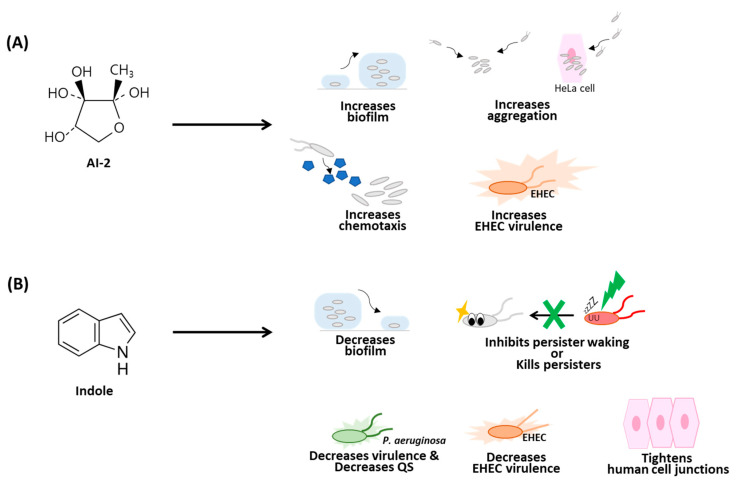
Comparison of the phenotypes affected by (**A**) autoinducer 2 (AI-2) and (**B**) indole. Curved black arrows indicate cell motility/movement, QS is quorum sensing, EHEC is *Escherichia coli* O157:H7, and flagella are indicated by two lines at one of the cell poles. Human cells are indicated by pink hexagons. Green lightning indicates the application of indole. The *R*-2-methyl-2,3,3,4-tetrahydroxytetrahydrofuran (*R*-THMF) form of AI-2 is shown.

Commensal *E. coli* has several QS pathways, including one system based on indole (Figure 1) [8,9,10], which is produced by TnaA from tryptophan, and another system based on autoinducer 2 (AI-2) (Figure 1) [11], which is produced by LuxS from *S*-ribosylhomocysteine [12]. It appears AI-2 is used primarily for communication inside the gastrointestinal tract at 37 °C, while indole is used primarily at lower temperatures (30 °C and lower) when the bacterium is outside of its eucaryotic host [9]. Although *E. coli* can detect homoserine lactones through the autoinducer-1 sensor SdiA (a LuxR homolog), it lacks a homoserine lactone synthase to produce the homoserine lactone signal, so *E. coli* uses SdiA to eavesdrop on signals of other bacteria [13]. Moreover, there is an interaction between these systems in that SdiA has been shown to be important for indole signaling in *E. coli* [8].

Once produced by LuxS, the AI-2 precursor 4,5-dihydroxy-2,3-pentanedione is converted spontaneously into *R*-2-methyl-2,3,3,4-tetrahydroxytetrahydrofuran (*R*-THMF) in *E. coli* (Figure 1), and *R*-THMF is the active form of AI-2 [7]. Hydrophilic AI-2 is transported from the cell by the membrane protein TqsA [14]. Once a threshold concentration is reached in the late exponential phase, AI-2 is imported into *E. coli* through its recognition by the AI-2 receptor LsrB [15]. In addition to LsrB in *E. coli*, LuxP (e.g., *Vibrio harveyi*) and the dCACHE-domain proteins PctA/TlpQ (*Pseudomonas aeruginosa*) are receptors for AI-2 [15], so there are at least three forms of AI-2 receptors in different bacteria. Furthermore, upon import, AI-2 is phosphorylated by LsrK in *E. coli*, and phosphorylated AI-2 binds and inhibits the repressor LsrR, which leads to changes in gene expression primarily at 37 °C [9].

## 3. AI-2 and Biofilm Formation

Although indole reduces both pathogenic [16] and non-pathogenic *E. coli* biofilm formation [17], AI-2 increases *E. coli* biofilm formation (Figure 1). Initially, QS was linked to biofilm formation using non-*E. coli* species and based on non-AI-2 signaling, specifically, for homoserine lactone increasing *Pseudomonas aeruginosa* [18]. Later studies, with *Vibrio cholerae* [19], *Serratia liquefaciens* [20], and *Streptococcus mutans* [21], confirmed the link of QS to biofilm formation.

The first report of AI-2 and biofilm formation was indirect and based on masking AI-2 signaling in *E. coli* with the QS inhibitor (*5Z*)-4-bromo-5-(bromomethylene)-3-butyl-2(*5H*)-furanone (henceforth furanone) from the alga *Delisea pulchra;* in this report, biofilm formation was reduced by 60 µg/mL furanone [22]. Later reports of AI-2 influencing biofilm formation were based on *luxS* mutants rather than purified AI-2. For example, a *luxS* mutation in *Streptococcus gordonii* influenced mixed-species biofilm formation with *Porphyromonas gingivalis* [23], a *luxS* mutation had a small impact on the architecture of *Klebsiella pneumoniae* (although there was no effect for a *luxS* mutant for intestinal colonization and colonization on polystyrene) [24], and a *luxS* mutant increased biofilm formation in *Helicobacter pylori* [25]. Unfortunately, these early results related to AI-2 via *luxS* mutations do not provide compelling evidence due to pleiotropic changes resulting from the *luxS* mutations.

The first direct demonstration that AI-2 was responsible for influencing biofilm formation was the 4- to 24-fold increase in biofilm formation in microtiter plates for three *E. coli* strains upon the addition of 11 µM of purified AI-2 [11]. Moreover, AI-2 failed to stimulate biofilm formation for an *lsrK* AI-2 regulation mutant, and AI-2 stimulated biofilm formation five-fold in flow cells [11]. A decade later, the Sourjik group rediscovered that AI-2 increases *E. coli* biofilm formation and extended the original results to show AI-2 increases aggregation through the adhesin antigen 43 and curli [26]. They [26] also confirmed that the AI-2 Lsr uptake/processing pathway influences *E. coli* biofilm formation [27].

## 4. AI-2 and Chemotaxis

The first indication that AI-2 affects *E. coli* motility was that the QS inhibitor furanone at 13 µg/cm^2^ inhibited *E. coli* swarming motility [22]; critically, the furanone also inhibited *E. coli* AI-2 signaling by 26,600-fold [22]. Next, furanone was shown to repress 44 of the 56 genes induced by AI-2, including those for chemotaxis (e.g., *aer, cheABRWYZ, tap, tsr, trg*) and motility (e.g., *motAB, flgABCDEFGHIJKLMN, fliACDFHIKLMNOPQ*) [28]. Therefore, AI-2 induces chemotaxis and motility genes in *E. coli*, and masking AI-2 signaling with furanone reduces motility and biofilm formation.

The first direct report of AI-2 as a chemoattractant for any species was the 2008 discovery that *Escherichia coli* O157:H7 (EHEC) is attracted to purified AI-2 [29]. For EHEC, AI-2 also increases both swimming motility and attachment to HeLa cells [29]. For non-pathogenic *E. coli*, microfluidic devices were used a year later to show AI-2 is an attractant [30]. Later, similar to their studies on biofilm formation, the Sourjik group confirmed that AI-2 attracts *E. coli* [26]. Furthermore, as with biofilms, indole signaling is opposite that of AI-2 since indole repels enterohemorrhagic EHEC [31], whereas AI-2 attracts EHEC [29] (Figure 1).

The mechanism by which AI-2 is detected in *E. coli* was determined to be the chemotactic receptor Tsr, which previously was known for its recognition of *L*-serine [32]; LsrB, the AI-2 receptor, was also shown to be necessary [32]. As with chemotaxis and biofilm formation, chemotaxis through Tsr was corroborated by the Sourjik group [26]. Furthermore, the Manson group also verified that AI-2 increases biofilm formation in *E. coli* and found that biofilm formation in this strain is enhanced by chemotaxis to AI-2 [33]. Therefore, AI-2 stimulates biofilm formation in *E. coli* by increasing aggregation and chemotaxis (Figure 1).

## 5. AI-2 and Virulence

The two main *E. coli* signals influence pathogens in an opposite manner—indole decreases EHEC chemotaxis, motility, biofilm formation, and adherence to epithelial cells at the physiologically relevant concentration of primarily 0.5 mM [31]; these results that indole decreases EHEC virulence were largely confirmed 12 years later by the Sperandio group [34,35] (Figure 1). Indole from *E. coli* also reduces the virulence of *P. aeruginosa* by masking its QS [36], prevents *P. aeruginosa* from resuscitating [37] from the dormant persister state [38], and tightens the epithelial cell junctions of the human host [39]. Indole and its derivatives also kill persister cells [40,41]. In contrast, AI-2 at 100 µM to 500 µM increases EHEC chemotaxis, motility, and adherence to epithelial cells and induces biofilm-related genes [29]. Moreover, AI-2 induces the expression of 23 genes of the locus of enterocyte effacement of EHEC [29]. Hence, in pathogenic *E. coli*, indole reduces pathogenicity, while AI-2 increases it.

## 6. Perspectives

The discovery that the *E. coli* AI-2 signal secreted by cells attracts other *E. coli* cells and leads to increased biofilm formation indicates that *E. coli* cells actively seek other *E. coli* cells to form communities [42]. Hence, it illustrates how bacteria can seek kin to increase their fitness, i.e., cells seek others to build communities (biofilms) to protect themselves from myriad stresses [43] and to increase their pathogenicity.

The chemoattractant property of AI-2 has also led to several synthetic biology applications. For example, biological nanofactories have been devised that detect and bind cancer cells and then produce AI-2 at the surface of the cancer cells, which attracts *E. coli* homing cells that internalize the synthesized AI-2 and then produce a biomarker or potentially an anti-cancer compound from an AI-2-induced promoter [44]. In this way, healthy cells could be discriminated from diseased ones. Therefore, the better understanding of the roles AI-2 and indole play in *E. coli* physiology has had a significant impact, both in our understanding of how communities are formed and in synthetic biology. Hence, AI-2 and indole are true and important signals in *E. coli*.
